# Transcriptomic and metabolomic profiling provide novel insights into fruit development and flesh coloration in Prunus mira Koehne, a special wild peach species

**DOI:** 10.1186/s12870-019-2074-6

**Published:** 2019-11-01

**Authors:** Hong Ying, Jian Shi, Shanshan Zhang, Gesang Pingcuo, Shuo Wang, Fan Zhao, Yongning Cui, Xiuli Zeng

**Affiliations:** 1 The ministry of agriculture of Qinghai-Tibet plateau fruit trees scientific observation test station, Lhasa, 850032 Tibet China; 2 Institute of Vegetables, Tibet Academy of Agricultural and Animal Husbandry Sciences, Lhasa, 850002 Tibet China; 3 Wuhan Metware Biotechnology Co., Ltd, Wuhan, 430070 China

**Keywords:** Gene regulatory network, Metabolites, Flesh pigmentation, Prunus mira

## Abstract

**Background:**

Flesh color is one of the most important traits for the commercial value of peach fruit. To unravel the underlying regulatory network in *Prunus mira*, we performed an integrated analysis of the transcriptome and metabolome of 3 fruit types with various flesh pigmentations (milk-white, yellow and blood) at 3 developmental stages (pit-hardening, cell enlargement and fruit ripening).

**Results:**

Transcriptome analysis showed that an intense transcriptional adjustment is required for the transition from the pit-hardening to the cell enlargement stage. In contrast, few genes were differentially expressed (DEGs) from the cell enlargement to the fruit ripening stage and importantly, the 3 fruits displayed diverse transcriptional activities, indicating that difference in fruit flesh pigmentations mainly occurred during the ripening stage. We further investigated the DEGs between pairs of fruit types during the ripening stage and identified 563 DEGs representing the ‘*core transcriptome*’ associated with major differentiations between the 3 fruit types, including flesh pigmentation. Meanwhile, we analyzed the metabolome, particularly, at the ripening stage and uncovered 40 differential metabolites (‘*core metabolome*’) between the 3 fruit types including 5 anthocyanins, which may be the key molecules associated with flesh coloration. Finally, we constructed the regulatory network depicting the interactions between anthocyanins and important transcripts involved in fruit flesh coloration.

**Conclusions:**

The major metabolites and transcripts involved in fruit flesh coloration in *P. mira* were unraveled in this study providing valuable information which will undoubtedly assist in breeding towards improved fruit quality in peach.

## Background

Prunus mira Koehne (syn. Amygdalus mira) is an important wild peach species native to China and widely distributed in the Tibetan Plateau [1]. It is a perennial fruit tree belonging to the family Rosaceae and the genus *Prunus*. In China, *P. mira* has special nutritional, economic, medicinal and ornamental values [2]. Its edible fruit is rich in nutrients (vitamin C, calcium, and iron) and fatty acids (oleic acid, linoleic acid, cetylic acid, and octadecanoic acid). Therefore, *P. mira* fruit and the processed fruit juice are commercialized, generating considerable economic profits [3]. Its fruit is also greatly employed in Chinese traditional medicine to treat irregular menstruations, fractures, and congestion owing to its high potential to enhance blood circulation [1]. The high content of arbutin in the fruit also promotes its use in the skin care industry. Moreover, *P. mira* flowers are recognized as presenting an ornamental interest [4]. As an ancestral species of many cultivated peach species including *Prunus persica*, *P. mira* represents a valuable reservoir of useful alleles for peach improvement [5]. At present, there are few systematic studies on the characteristics and quality of *P. mira* fruit. In contrast to the research on its cultivation which started very early, molecular research was instigated late, explaining the lack of progress in the exploitation and valorization of *P. mira* germplasm resources in China [6].

Flesh color is one of the most important traits for the commercial value of peach fruit which has implications for consumer acceptance and nutritional quality [7, 8]. There is a rich natural variation in Tibetan *P. mira* fruit flesh color [9]. Knowledge of the genetic basis of characters related to Tibetan *P. mira* fruit quality such as the flesh color will facilitate their manipulation to obtain more attractive and healthier fruit for the consumer [10]. Three main classes of accessory pigments, namely, flavonoids, caretonoids, and betalains have been identified in plants [11]. Flavonoids, particularly anthocyanins, provide a wide range of colors ranging from orange/red to violet/blue. The biosynthetic pathway of anthocyanin has been well-characterized in fruit trees [12]. Various structural genes (phenylalanine ammonia-lyase (PAL), chalcone synthase (CHS), chalcone isomerase (CHI), flavonone 3-hydroxylase (F3H), dihydroflavonol 4-reductase (DFR), anthocyanin synthase (ANS), and UDP-glucose-flavonoid 3-*O*-glucosyltrasnferase (UFGT) and transcription factors such as MYB, basic helixloop-helix (bHLH) and WD40 genes were reported to be key determinant of anthocyanin biosynthesis and accumulation [13, 14]. Betalains are found only in the order Caryophyllales and substitute for anthocyanins [12]. Concerning the carotenoids, they are a class of terpenoids involved in plant photoprotection and coloration [15]. Depending on the concentration and types, carotenoid-rich plant organs can show a wide spectrum of colors including yellow, orange and red [16]. Previous studies in peach species suggested that the red fruit coloration is determined by the content and composition of anthocyanins while the yellow color is associated to carotenoids [17–21]. In addition, it was demonstrated that pigment (anthocyanin and carotenoid) accumulation is dependent on the fruit developmental stages [18, 21, 22]. In the cultivated peach (*P. persica* L. Batsch), the high activity of the gene *ccd4* leading to a low carotenoid level was pinpointed as determinant for the white-flesh phenotype as opposed to the yellow-flesh one [18, 19]. By studying the blood-flesh and white-flesh peach, Jiao et al. [20] showed that *PAL* gene was weakly expressed in the white-flesh peach and proposed that *PAL* gene may be limiting anthocyanin production, leading to the white-flesh coloration.

In recent years, high-throughput methods have been extensively applied to understand organ coloration in plants. Integrated metabolomic and transcriptomic analyses in plant organs shed light on the relationship between the contents of various secondary metabolites and the corresponding differentially expressed genes [23–25]. Combining transcriptome and metabolome approaches, Wang et al. [25] revealed that the red color fading of ‘Red Bartlett’ (*Pyrus communis*) cultivar was closely linked to the reduced anthocyanin biosynthesis, the increased anthocyanin degradation and the suppression of anthocyanin transport. Another important study in fig (*Ficus carica* L.) reported that the anthocyanin metabolite cyaniding O-malonylhexoside increased 3992-fold in the purple peel cultivar compared to the green peel cultivar, similarly as various other anthocyanin pathway components [26]. By analyzing the gene expression change, they observed that the high accumulation of anthocyanin compounds was correlated with an up-regulation of genes encoding flavonoid and anthocyanin pathway components and transcription factors such as R2R3-MYB in the purple peel cultivar mainly at the mature stages. Recently, Li et al. [22] also identified 7 flavonoids metabolites (bracteatin, luteolin, dihydromyricetin, cyanidin, pelargonidin, delphinidin and (−)-epigallocatechingenes) and six genes (*AaF3H*, *AaLDOX*, *AaUFGT*, *AaMYB*, *AabHLH*, and *AaHB2*) as the best candidates involved in the pigmentation of all-red-fleshed and all-green-fleshed in *Actinidia arguta*.

In the present work, we investigated for the first time *P. mira* fruit flesh coloration in relation to the developmental stages using integrated transcriptomic and metabolomic analyses. Fruit from two wild trees: PMHR (blood-colored flesh), PMHY (yellow-colored flesh) and a semi-wild tree PMHF (milk-white colored flesh), were harvested at the pit-hardening, cell enlargement and fruit ripening stages. This study aimed at assessing the metabolic pathways and candidate genes underlying the various flesh colorations. On the other hand, we explored the dynamism of the transcriptome and metabolome changes at different fruit development stages. Our results provide further insights into fruit development and flesh coloration in plants, shed light on the enormous chemical diversity in *P. mira* fruit and will undoubtedly assist in efforts to improve fruit appeal and quality in peach species.

## Results

### Overview of the fruit transcriptome sequencing in *P. mira*

**Fig. 1:**
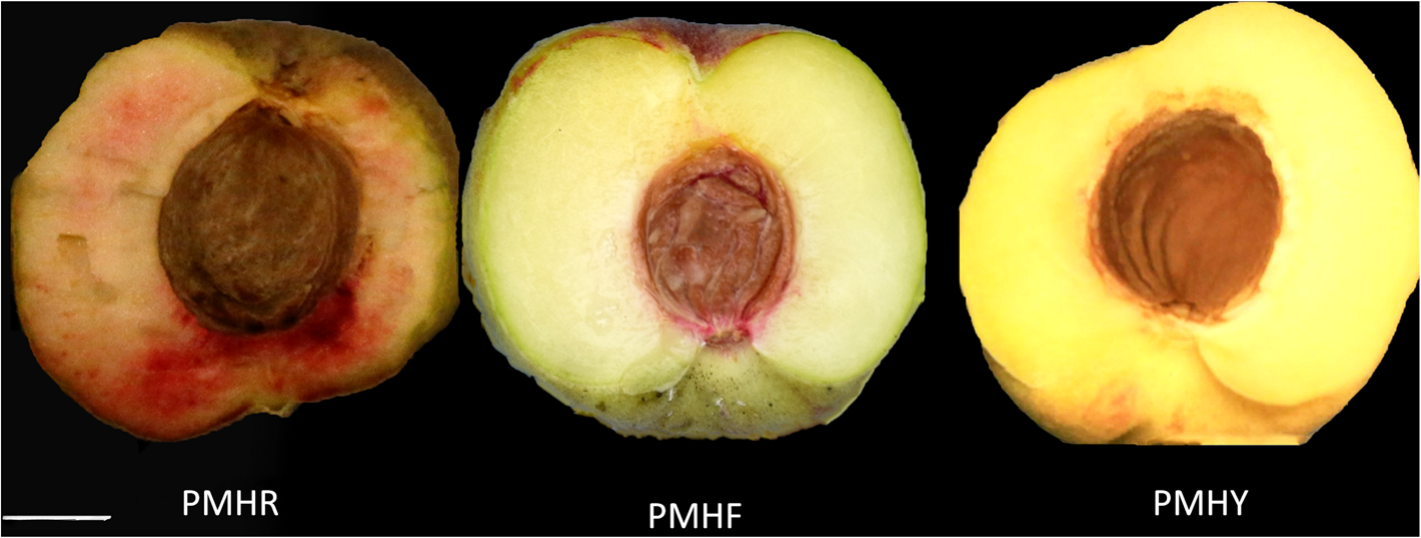
The phenotypes of the fruit flesh of *Prunus mira* cv. “PMHR”, “PMHF” and “PMHY”. Bar = 1 cm

**Fig. 2:**
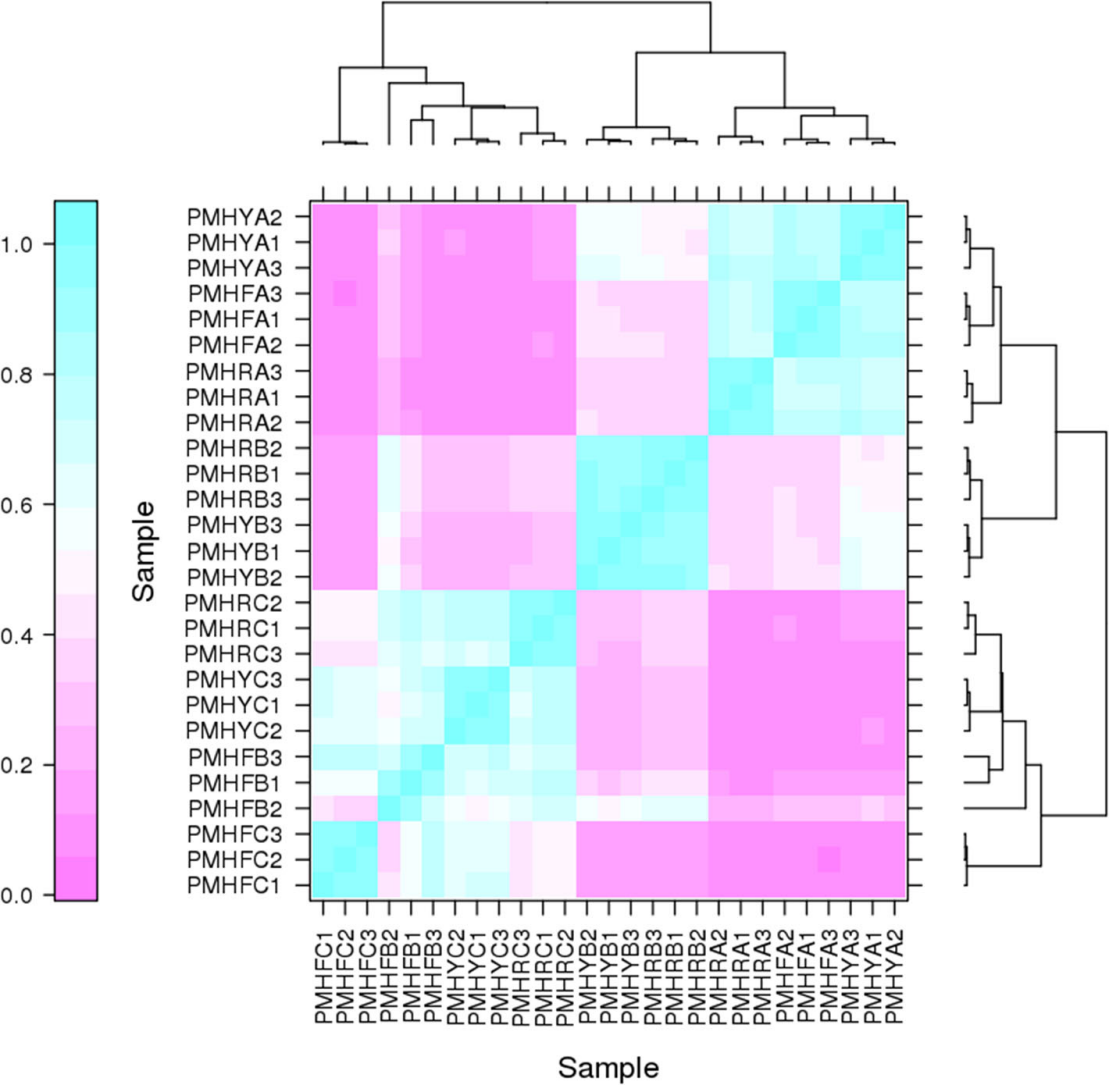
Heatmap clustering showing correlation among *Prunus mira* different samples based on global expression profiles

Three types of *P. mira* fruit (PMHY, PMHR and PMHF) with various flesh colorations were collected at different development stages: pit hardening (A), cell enlargement (B) and fruit ripening (C) and used to construct nine sequencing libraries named PMHYA, PMHYB, PMHYC, PMHRA, PMHRB, PMHRC, PMHFA, PMHFB and PMHFC (Fig. 1). With 3 biological replicates, transcriptome sequencing of the 27 samples yielded a total of 195.56 Gb clean data with 92.21% of bases scoring Q30 and above (Additional file 1: Table S1). Of the total clean reads, 75.44 to 84.41% were unique matches with the *P. persica* reference genome (ftp://ftp.ncbi.nlm.nih.gov/genomes/all/GCF/000/346/465/GCF_000346465.2_Prunus_persica_NCBIv2/GCF_000346465.2_Prunus_persica_NCBIv2_genomic.fna.gz). A total of 17,533 expressed genes in PMHFA, 17,725 in PMHFB, 17,248 in PMHFC, 17,524 in PMHRA, 17,719 in PMHRB, 17,531 in PMHRC, 17,291 in PMHYA, 17,671 in PMHYB, 17,740 in PMHYC were detected, resulting in 23,809 unique genes in *P. mira* (Additional file 2: Table S2). Based on the novel gene excavation analysis, a total of 1054 genes were specific to *P. mira*, 864 of which were functionally annotated (Additional file 3: Table S3). However, it is probable that these genes do exist in peach genome and were not detected by the prediction algorithm used for the reference set of genes. In addition 4037 genes were optimized based on the gene structure optimization analysis in *P. persica* (Additional file 4: Table S4, Additional file 5: Table S5). Hierarchical clustering of the samples based on the number of fragments per kilobase of exon per million fragments mapped (FPKM) showed that all the biological replicates clustered together, suggesting a high reliability of our sequencing data (Fig. 2). Furthermore, it could be observed that global gene expression varied distinctly from one fruit development stage to another but the variation according to the flesh color was insignificant. This result implies that the expression of a large number of genes is altered during fruit development whereas only a few genes are associated with the differences in fruit flesh coloration in *P. mira*.

### Change in gene expression during fruit developmental stages in *P. mira*

**Fig. 3:**
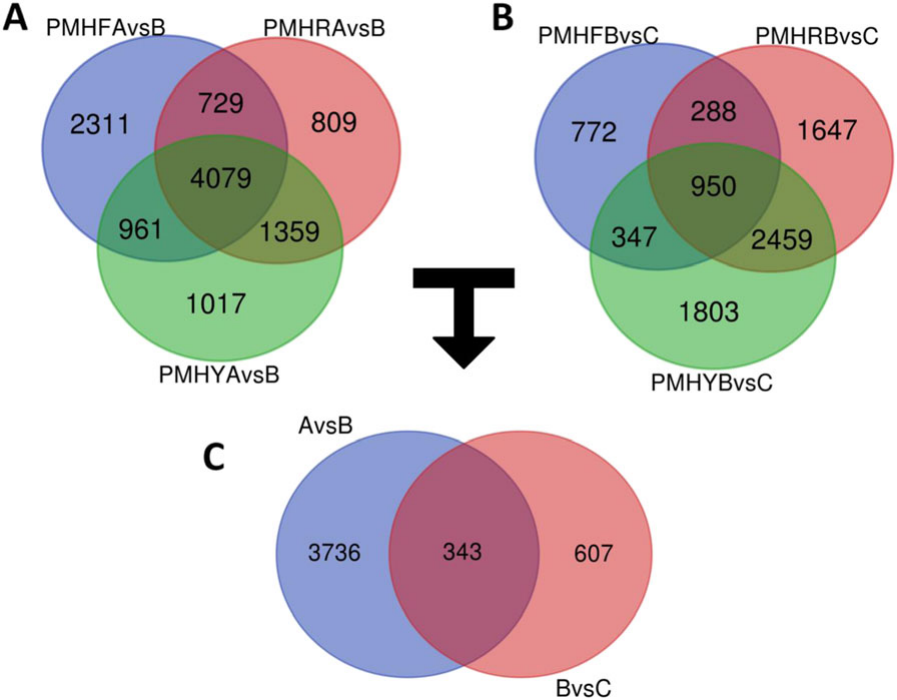
Differential expressed genes (DEG) during fruit developmental stages in *Prunus mira*. a. Venn diagram showing the shared and unique DEGs between pit-hardening (A) and cell enlargement (B) stages in PMHF, PMHR and PMHY; **b**. Venn diagram showing the shared and unique DEGs between cell enlargement (B) and fruit ripening (C) stages in PMHF, PMHR and PMHY; **c**. Venn diagram showing the shared and unique DEGs between the transitions from A to B and B to C

To uncover the main genes differentially expressed over the different fruit development stages and those specific to the transition from the pit hardening to the cell enlargement stages (AvsB) and subsequently to the fruit ripening stage (BvsC), we cross-compared the differential expressed genes (DEGs) in PMHF, PMHY, PMHR and sorted out the shared DEGs (Additional file 6: Table S6). For AvsB, we detected 8080, 7416 and 6976 DEGs in PMHF, PMHY and PMHR, respectively, with 4079 DEGs commonly identified in the three fruit types (Fig. 3a). Regarding BvsC, we identified 2357, 5344 and 5559 DEGs in PMHF, PMHY and PMHR, respectively, of which, only 950 DEGs were common to the 3 fruit types (Fig. 3b). These results indicate that the molecular mechanisms occurring at the pit hardening and cell enlargement stages are quite well conserved among the different fruit types but the fruit ripening process involves differential molecular mechanisms in the various fruit types. Accordingly, the fruit ripening stage should be targeted to get insights into the altered pathways and genes associated with the fruit flesh coloration in *P. mira*. As shown in Fig. 3c, 343 genes (enriched in KEGG pathways related to MAPK signaling pathway, starch and sucrose metabolism, steroid biosynthesis, carotenoid biosynthesis, etc. (Additional file 7: Figure S1)), were constantly and differentially expressed during the transitions from A to B and B to C, denoting that they represent the main genes associated to fruit development in *P. mira*, regardless of the fruit type. Meanwhile, we obtained 3736 genes (enriched in KEGG pathways related to MAPK signaling pathway, amino sugar and nucleotide sugar metabolism, biosynthesis of antibiotics, starch and sucrose metabolism, amino sugar and nucleotide sugar metabolism, etc. (Additional file 8: Figure S2)) and 607 genes (enriched in KEGG pathways related to plant hormone signaling pathways, MAPK signaling pathways, cysteine and methionine metabolism, etc. (Additional file 9: Figure S3)) specific to the transition from A to B and B to C, respectively. A total of five genes were randomly selected within the DEGs list and their FPKM fold changes in the three genotypes at the three fruit developmental stages were well correlated with the relative expression via qRT-PCR (*R*^2^ = 0.74; Additional file 10: Figure S4).

### Identification of differential expressed genes related to fruit flesh coloration

**Fig. 4:**
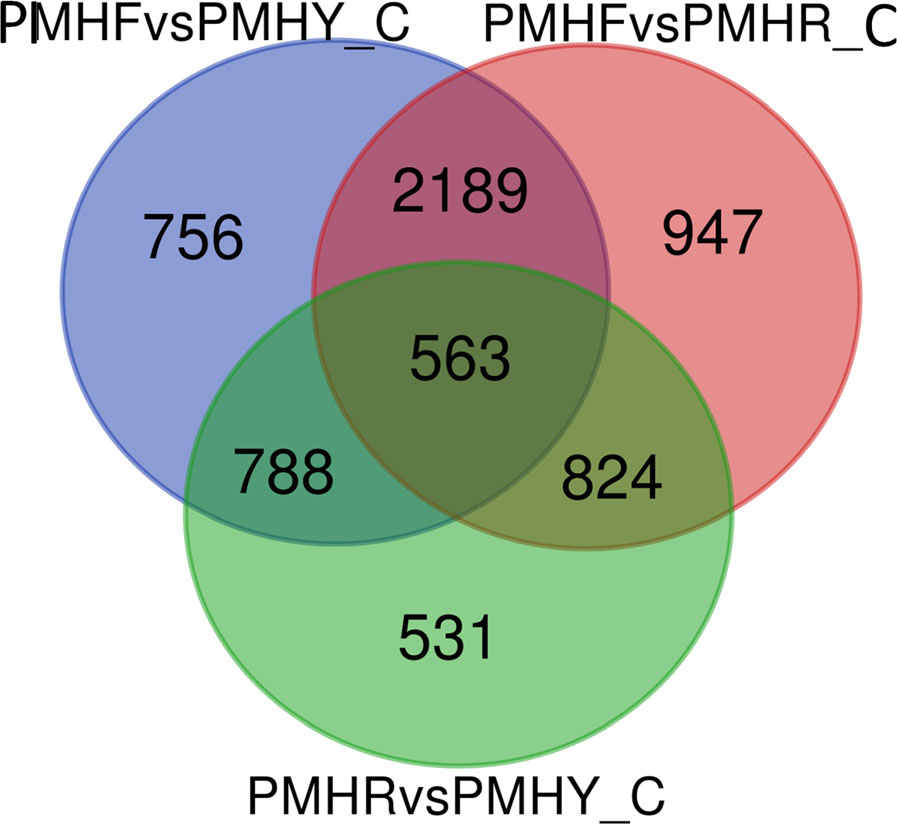
Identification of the shared and unique differential expressed genes between pairs of fruit types (PMHFvsPMHR, PMHFvsPMHR, PMHRvsPMHY) at the fruit ripening stage (C) in *Prunus mira*

**Fig. 5:**
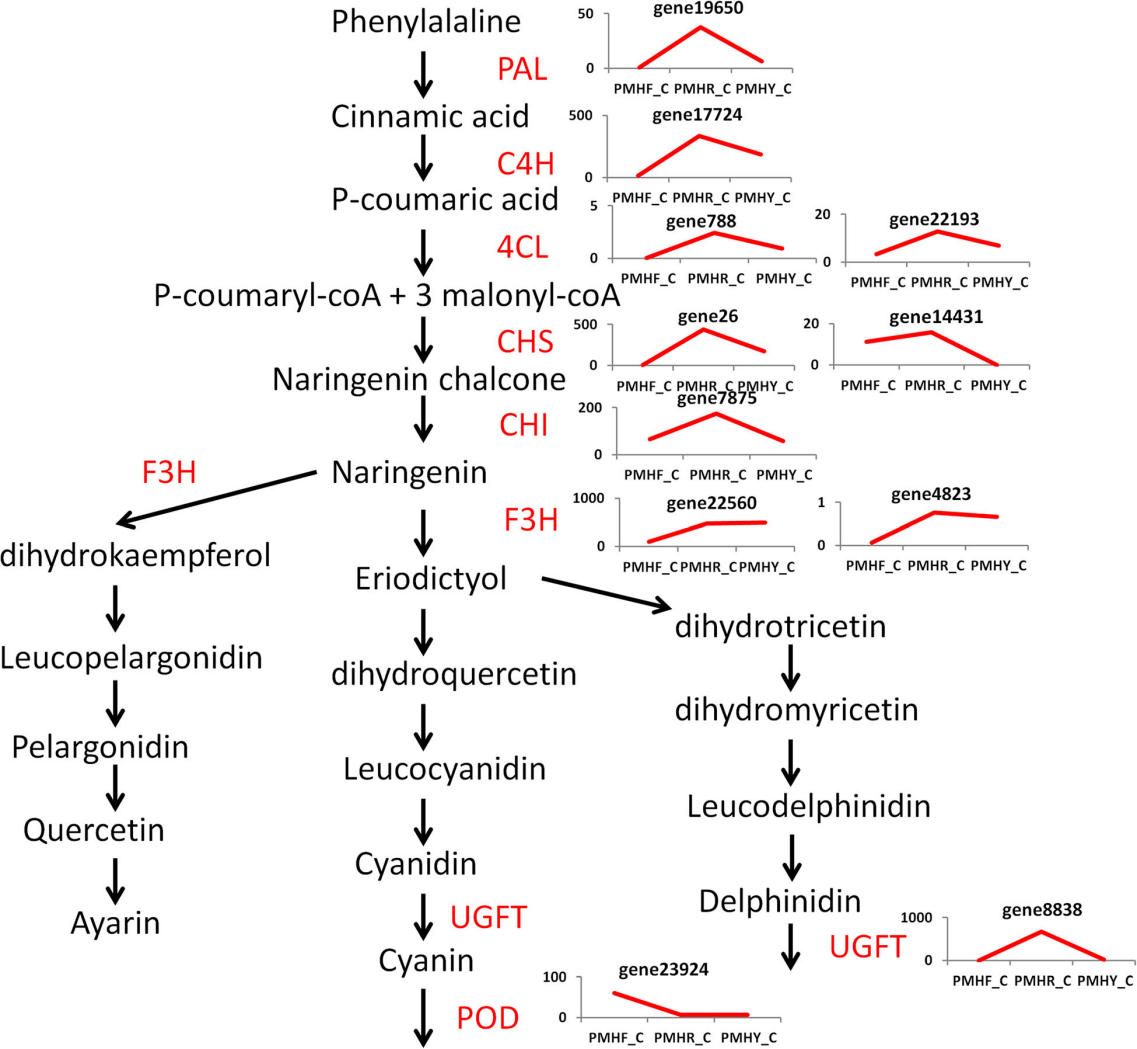
Phenylpropanoid-flavonoid biosynthetic pathway-related genes differentially expressed in PMHF, PMHR and PMHY during the fruit ripening stage (C). Phenylalanine ammonia-lyase (PAL), cinnamic acid 4-hydroxylase (C4H), 4 coumarate CoA ligase (4CL), chalcone synthase (CHS), chalcone isomerase (CHI), flavanone 3-hydroxylase (F3H), UDP-flavonoid glucosyl transferase (UFGT) and peroxidase (POD). The line chart displays the expression levels of the genes (FPKM value)

To identify the DEGs related to fruit flesh coloration, we compared the FPKM values of PMHF to PMHY (milk white -colored flesh compared to yellow-colored flesh), PMHF to PMHR (milk white -colored flesh compared to blood-colored flesh) and PMHR to PMHY (blood-colored flesh compared to yellow-colored flesh) at the fruit ripening stage (C). For PMHFvsPMHY, we detected a total of 4296 DEGs including 2094 down- and 2202 up-regulated genes. Similarly, we analyzed the DEGs between PMHFvsPMHR and obtained a total of 4523 DEGs including 2387 down- and 2136 up-regulated genes. Concerning PMHRvsPMHY, we found fewer amounts of DEGs (2706) including 1142 down- and 1564 up-regulated genes. We deduced that change from milk-white to blood or yellow coloration may require a larger transcriptional reconfiguration than change from yellow to blood coloration. Next, we compared the DEGs identified in PMHFvsPMHY, PMHFvsPMHR and PMHYvsPMHR to pinpoint those common the three color change schemes. This resulted in 563 genes constitutively and differentially expressed between the 3 fruit types implying that they may represent the ‘*core transcriptome*’ associated to major differentiations between the 3 fruit types, including flesh coloration (Fig. 4, Additional file 11: Table S7). They were enriched in KEGG pathways related to plant hormone signal transduction, pentose and glucuronate interconversions, starch and sucrose metabolism, metabolism of xenobiotics by cytochrome P450, etc. (Additional file 12: Figure S5). Within these genes, only 6 DEGs (*gene16639*, *gene14633*, *gene4519*, *gene9018*, *gene18806* and *gene24803*) are involved in the terpernoid-carotenoid pathways while many genes (*gene17724*, *gene788*, *gene22193*, *gene13700*, *gene23130*, *Prunus_persica_newGene_904*, *gene23924*, *gene19650*, *gene26*, *gene14431*, *gene22560*, *gene7875*, *gene4823* and *gene21971*) participate in the phenylpropanoid-flavonoid pathways (Fig. 5, Additional file 13: Table S8), suggesting that structural genes in the phenylpropanoid-flavonoid pathways are the main regulators of fruit flesh pigmentation in *P. mira*. In addition, analysis of the transcription factor families including MYB, basic-Helix-Loop-Helix (bHLH) and WD40 which modulate the expression level of flavonoid biosynthetic structural genes unraveled 5 MYBs (*gene20317*, *gene9054*, *gene25173*, *gene13863* and *gene22870*), 3 bHLHs (*gene17547*, *gene19413* and *gene6656*) but no WD40 (Additional file 14: Table S9). Within the MYB regulators, 4 genes (*gene20317*, *gene9054*, *gene25173* and *gene22870*) were highly expressed in colored flesh fruits (PMHR and PMHY) than PMHF while only the gene *gene13863* showed the opposite trend. Similarly, concerning the bHLH transcription factors, only the gene *gene19413* was more expressed in PMHF compared to PMHR and PMHY. A total of five genes were randomly selected within the DEGs list and their FPKM fold changes in the three genotypes at the three fruit developmental stages were well correlated with the relative expression via qRT-PCR (*R*^2^ = 0.74; Additional file 10: Figure S4).

### Overview of the fruit flesh metabolome profiling in *P. mira*

**Fig. 6:**
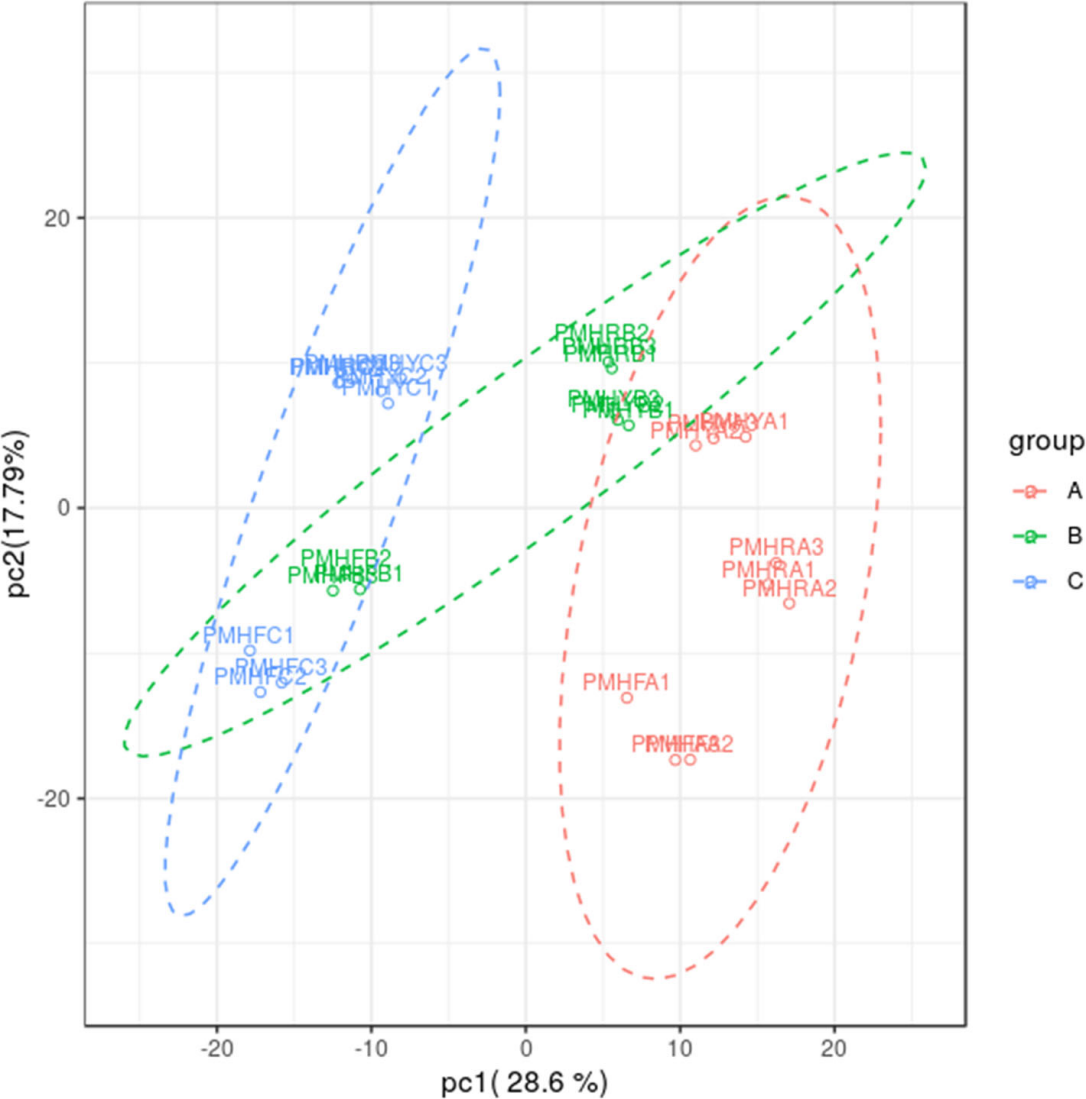
Principal component analysis of the metabolite quantification in 3 *Prunus mira* fruit types (PMHF, PMHR and PMHY) at 3 fruit developmental stages (pit-hardening (A), cell enlargement (B) and fruit ripening (C))

We further analyzed the metabolic alteration in *P. mira* fruit during the 3 developmental stages using a widely-targeted approach. In total, 493 compounds were successfully detected and determined for the first time in *P. mira* (Additional file 15: Table S10). The diversity and richness of the metabolites detected in this work may represent an excellent opportunity to identify novel compounds associated with fruit development, coloration and quality. The identified compounds could be classified into 33 classes, predominantly, organic acids, nucleotide and its derivatives, and amino acid derivatives (Table 1). Principal component analysis (PCA) of the metabolic quantification from the 3 fruit types showed that all biological replicates were grouped together, which indicates a good correlation between replicates and the high reliability of our data. Moreover, we observed that at each stage (A, B, and C), the metabolites from PMHF were obviously distinct from those of PMHR and PMHY (Fig. 6). A separation between the developmental stages could be observed by the PC1 while a separation according to the fruit types by the PC2 could be observed. Overall, these results are consistent with the transcriptional responses between the 3 fruit types.

**Table 1:** Classification of the 493 detected metabolites into major classes

Class	Number of compound	Class	Number of compound	Class	Number of compound
Flavonolignan	1	Alcohols and polyols	7	Carbohydrates	18
Pyridine derivatives	1	Flavone C-glycosides	7	Quinate and its derivatives	20
Terpenoids	1	Phenolamides	7	Flavone	21
Isoflavone	2	Catechin derivatives	9	Flavonol	22
Alkaloids	3	Benzoic acid derivatives	10	Amino acids	26
Nicotinic acid derivatives	3	Phytohormones	11	Others	26
Tryptamine derivatives	3	Coumarins	12	Hydroxycinnamoyl derivatives	27
Cholines	4	Vitamins	13	Lipid_Glycerophospholipid	28
Proanthocyanidins	4	Flavanone	15	Amino acid derivatives	45
Anthocyanins	5	Lipid_Glycerolipid	15	Nucleotide and its derivates	47
Indole derivatives	6	Lipid_Fatty acids	16	Organic acids	58

### Analysis of differentially altered metabolites in relation to fruit flesh coloration

Since the fruit ripening stage was identified as the key period for flesh color determination in *P. mira* based on the transcriptome results, we scrutinized the differentially accumulated metabolites (DAM) during this fruit developmental stage in PMHF compared to PMHY (PMHFvsPMHY), PMHF compared to PMHR (PMHFvsPMHR) and PMHR compared to PMHY (PMHRvsPMHY). The differential metabolites between group comparisons were identified based on the fold-change and the variable importance in projection (VIP).

**Fig. 7:**
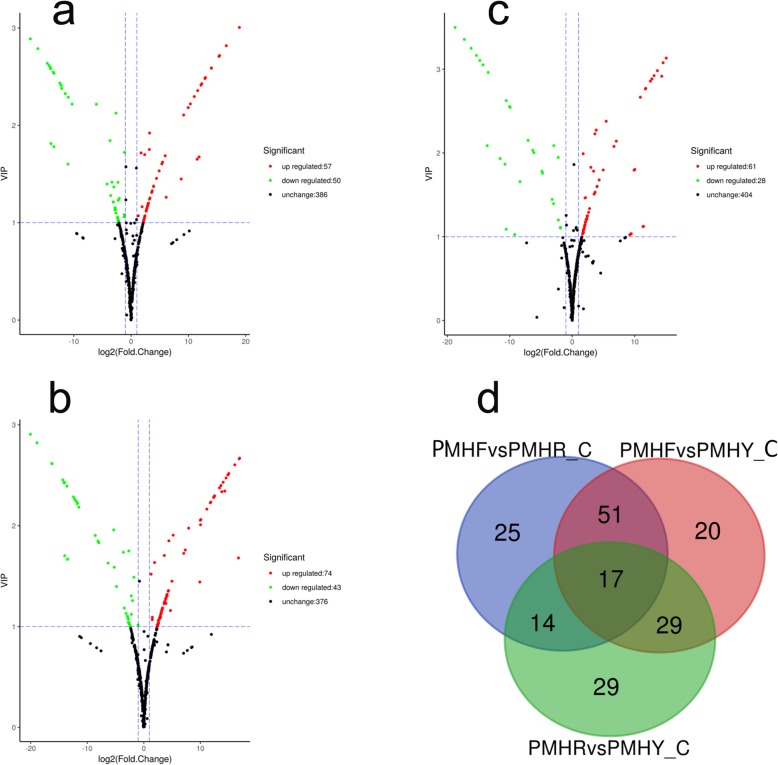
Identification of the differentially accumulated metabolites (DAM) between pairs of fruit types during the fruit ripening stage. **a**. PMHFvsPMHR, **b**. PMHFvsPMHY, **c**. PMHRvsPMHY, **d**. Venn diagram depicting the shared and unique DAMs between group comparisons

**Fig. 8:**
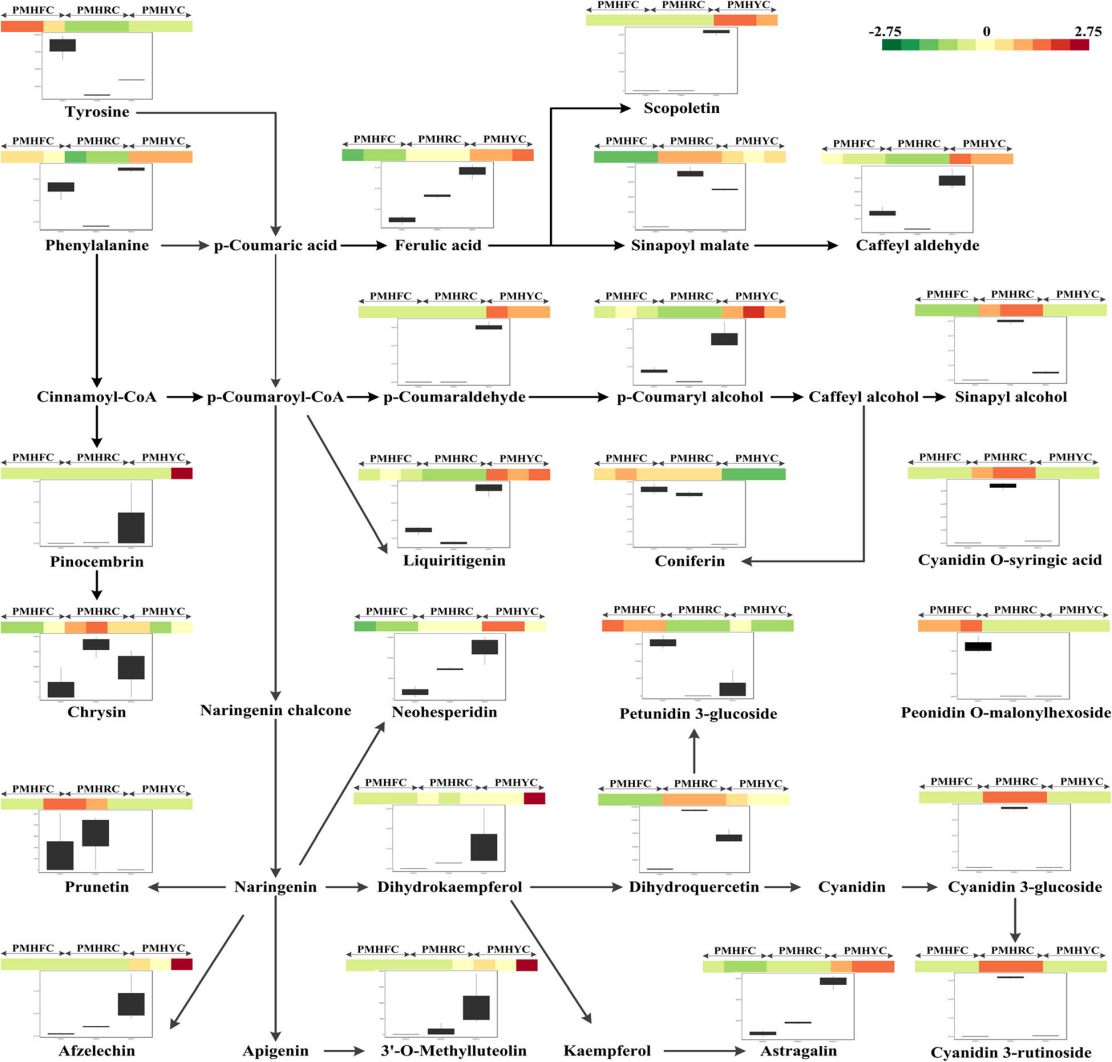
Overview of the phenylpropanid-flavonoid biosynthetic pathway during the fruit ripening stage in 3 *Prunus mira* fruit types (PMHF, PMHR and PMHY). Color scale from dark green to dark red for the heatmap represents the log2 value of the metabolite content. The boxplots based on 3 biological replicates show the variation of the metabolite contents in the 3 fruit types

For PMHFvsPMHY, we found 117 DAMs of which, 43 and 74 were down- and up-accumulated compounds, respectively (Fig. 7a). Similarly, for PMHFvsPMHR, a total of 107 DAMs including 50 down- and 57 up-accumulated metabolites were detected (Fig. 7b). We identified 89 DAMs including 27 down- and 62 up-accumulated compounds in PMHRvsPMHY (Fig. 7c). As shown in Fig. 7d, 40 diverse metabolites were commonly shared by the 3 color change schemes, which may constitute the ‘*core metabolome*’ associated to major differentiations between the 3 fruit types of *P. mira* (Additional file 16: Table S11). Within these compounds, 25 were mapped to the phenylpropanoid-flavonoid pathways (Fig. 8) and 5 anthocyanins (Jur80, Jur487, Jur861, Jur1147 and Jur929) were detected which may potentially be the key molecules modulating fruit flesh pigmentation in *P. mira* (Table 2).

**Table 2:** List of the anthocyanins identified in the ‘*core metabolome*’ regulating flesh coloration in the 3 fruit types and their Log_2_ fold change values

Compound	Name	Class	PMHF vs PMHR	PMHF vs PMHY	PMHR vs PMHY
Jur80	Cyanidin 3-O-rutinoside (Keracyanin)	Anthocyanins	9.17	3.04	−6.13
Jur487	Petunidin 3-O-glucoside	Anthocyanins	−14.06	−2.64	11.42
Jur1147	Cyanidin O-syringic acid	Anthocyanins	18.89	14.03	−4.86
Jur929	Cyanidin 3-O-glucoside (Kuromanin)	Anthocyanins	15.34	2	−13.34
Jur861	Peonidin O-malonylhexoside	Anthocyanins	−13.59	−11.59	−2

### Regulatory network depicting interactions between important anthocyanins and transcripts associated with fruit flesh coloration in *P. mira*

**Fig. 9:**
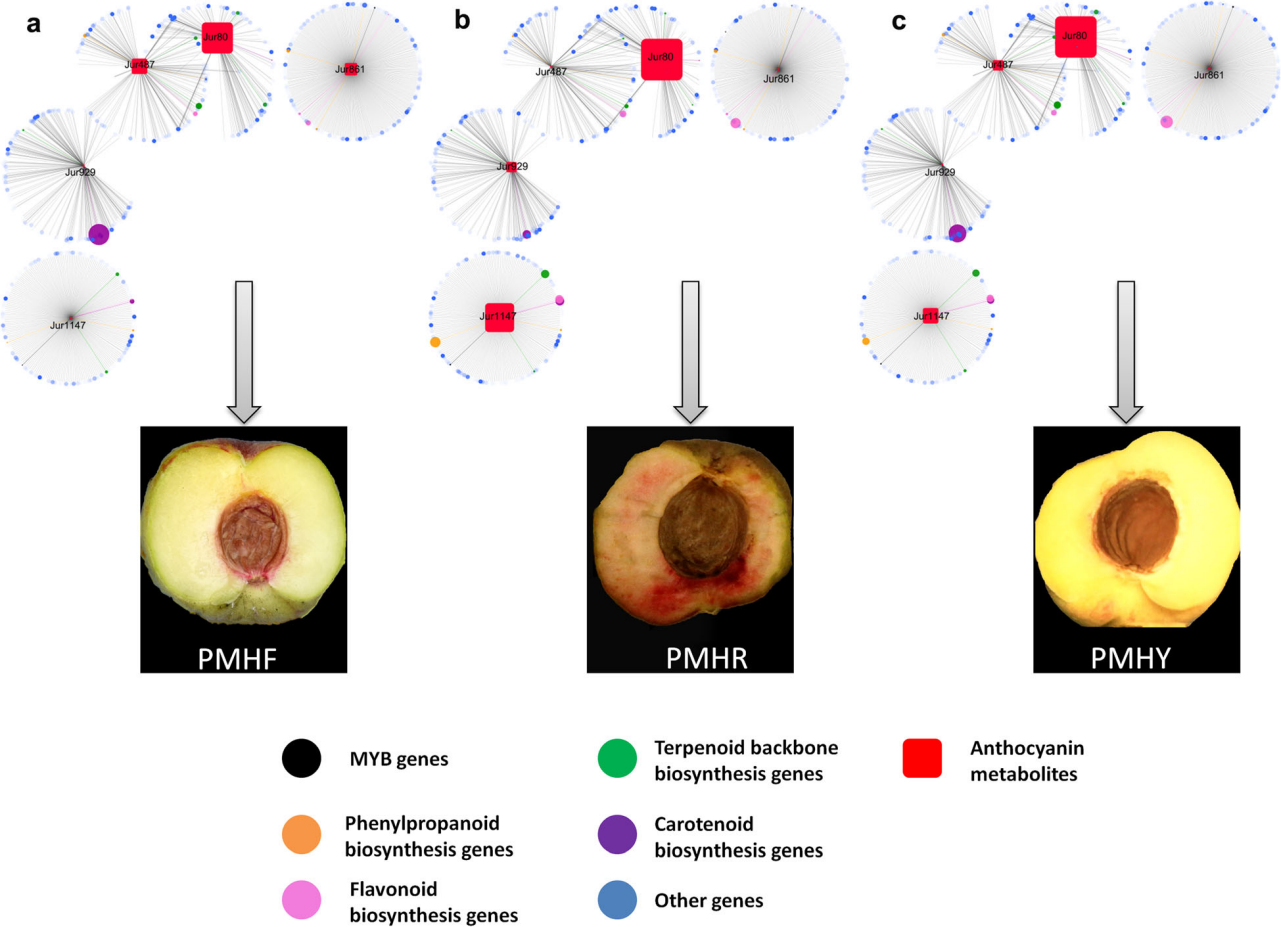
Regulatory networks connecting transcripts and 5 anthocyanin-related metabolites. The networks in **a.** PMHF, **b.** PMHR, **c.** PMHY were visualized with the Cytoscape software (version 3.6.1)

To understand the regulatory network of anthocyanins involved in the differential pigmentation of the 3 colored *P. mira* fruit, we carried out correlation tests between quantitative changes of the 5 anthocyanins from the ‘*core metabolome*’ and transcripts from the ‘*core transcriptome*’. The result showed that 183, 328, 213, 228 and 159 transcripts had strong correlation values (*R*^2^ > 0.9) with Jur487, Jur861, Jur1147, Jur929 and Jur80, respectively (Additional file 17: Table S12). The metabolites Jur80, Jur929 and Jur487 appeared to be more closely related than the other compounds and also shared many similar associated transcripts (Fig. 9). The regulatory network highlighted the major transcripts associated with the anthocyanins and more importantly, the key anthocyanin compounds relevant to each flesh color type. For example, Jur861 (peonidin O-malonylhexoside) was highly accumulated in PMHF while only traces of this metabolite could be detected in PMHR and PMHY, suggesting that Jur861 is important for the milk-white flesh coloration. By analyzing the associated transcripts, the gene *gene22560* (naringenin 3-dioxygenase) had the highest *R*^*2*^ score (0.98) and was up-regulated in PMHR and PMHY compared to PMHF (Log_2_foldchange values = 2.39, 2.46, respectively). This indicates that a weak expression of *gene22560* may partly promote peonidin O-malonylhexoside over-accumulation which is associated with the milk white -colored flesh fruit type in *P. mira*. With regard to the compound Jur929 (cyanidin 3-O-glucoside (kuromanin)), it was strongly accumulated in PMHR whereas, only traces could be detected in PMHF and PMHR, indicating that kuromanin is a key molecule for the blood pigmentation in *P. mira* fruit flesh. The gene *gene9018* (phytoene synthase) had the highest correlation score (0.98) with Jur929 but its expression level was significantly low in PMHR compared to PMHY and PMHF (Log_2_fold change values = 1.05, 1.07, respectively). This result implies that *gene9018* may be involved in a negative regulation of kuromanin synthesis in *P. mira* fruit. The compound Jur1147 (cyanidin O-syringic acid) is only present in the PMHR and PMHY but in a higher quantity in PMHR. From the regulatory network, 4 genes (*gene24803* ((+)-abscisic acid 8′-hydroxylase), *gene17724* (trans-cinnamate 4-monooxygenase), *gene21971* (beta-glucosidase) and *gene26* (chalcone synthase)) were remarkably important for the accumulation of Jur1147. Analysis of their expression levels showed that they are positively associated with the accumulation of Jur1147 and their higher activities in PMHR could partly explain the higher content of Jur1147. Concerning the metabolite Jur487 (petunidin 3-O-glucoside), it was only detected in the fruit flesh of PMHF and PMHY, though, at a lesser quantity in PMHY. Two genes *gene7875* (chalcone isomerase) and *gene16639* (1-deoxy-D-xylulose-5-phosphate reductoisomerase) from the regulatory network showed high correlation scores with Jur487. The gene *gene7875* was negatively associated with the accumulation of Jur487 while *gene16639* showed the opposite trend. Meanwhile, the only metabolite accumulated in a high quantity in all the 3 fruit types was Jur80 (cyanidin 3-O-rutinoside (keracyanin)). However, the exact quantification of keracyanin in the 3 fruit types showed that Jur80 was more accumulated in PMHR (3.17E^+ 08^) than PMHY (4.52E^+ 06^) and PMHF (5.49E^+ 05^), denoting that abundant accumulation of Jur80 tends to darken the fruit flesh color, from white to blood.

Overall, the blood coloration may be a combination of a high content of Jur80, Jur1147 and Jur929; the yellow color seemed to result from a moderate accumulation of Jur80, Jur1147 and Jur487; while the milk-white color could be formed thanks to the weak accumulation of Jur80 and a high content of Jur487 and Jur861 in *P. mira* fruit flesh.

## Discussion

In the present study, an integrated analysis of the transcriptome and metabolome was conducted to elucidate the fruit flesh coloration in *Prunus mira*, which is an important quality trait in peach [8]. By using the reference genome of *Prunus persica* [27], we observed a high conservation of the gene models in both species, although 1054 genes were found specific to *P. mira*. These specific genes may represent important wild resources for the improvement of the cultivated peach towards abiotic stress tolerance, fruit quality, disease resistance, etc. [28]. We also provide here for the first time extensive metabolome datasets from *P. mira* fruit which could fuel future studies on fruit quality traits in wild and cultivated peach species [29].

During the fruit development, the complex network of metabolites and proteins is dramatically altered [30]. Transcriptome and metabolome analyses over 3 fruit developmental stages in *P. mira* indicated that different molecular programs are involved in the transition from the pit-hardening to the cell enlargement stage and subsequently from the cell enlargement to the ripening stage. Previously, Lombardo et al. [29] also reported similar findings in the cultivated peach. They showed that high levels of bioactive polyphenols and amino acids, which are substrates of phenylpropanoid and lignin pathways, are important during pit hardening while a decrease in amino acid levels and an induction of transcripts encoding sucrose degradative and synthetic enzymes, are involved in ripening. In-depth analyses of the specific genes and metabolites detected for each fruit developmental stage will illuminate the biological processes underlying fruit growth and ripening in *P. mira*.

By comparing the transcriptional activity between fruit developmental stages in the 3 fruit types, we observed that they diverged principally during the ripening stage, denoting that major differences in the fruit phenotypes such as flesh pigmentation occur during this stage. Consistent with our report, Roca et al. [31] showed that carotenoids which are involved in plant pigmentation were found differentially accumulated only during ripening in different olive varieties. Furthermore, by analyzing peach fruit for their anthocyanin contents (which are also important for fruit color formation), Cao et al. [32] observed that the flesh anthocyanin contents were mainly differentially accumulated at the early ripening stage in various colored fruit types.

Flavonoids and caretonoids represent the major molecules involved in pigmentation in plants [11]. In this work, flavonoids, particularly, anthocyanins appeared to be more important in *P. mira* fruit flesh pigmentation than carotenoids. This was underscored by the fact that a high number of transcripts belonging to the phenylpropanoid-flavonoid pathways in the ‘*core transcriptome*’ and the majority of metabolites detected in the ‘*core metabolome*’ were mapped to the phenylpropanoid-flavonoid pathways. The anthocyanin biosynthetic pathway is a specific branch of the flavonoid pathway, which starts from the key amino acid phenylalanine to produce 4-coumaroyl CoA by phenylalanine ammonia-lyase (PAL), cinnamic acid 4-hydroxylase (C4H) and 4 coumarate CoA ligase (4CL) [33]. The main precursors for flavonoids are 4-coumaroyl CoA and three molecules of malonyl CoA that produce chalcones by chalcone synthase (CHS) [34]. Then, the pathway is catalyzed by a number of enzymes to yield flavanones (via chalcone isomerase, CHI), dihydroflavonols (via flavanone 3-hydroxylase, F3H), leucoanthocyanidins (via dihydroflavonol 4-reductase, DFR), anthocyanidins (via leucoanthocyanidin dioxygenase, LDOX) and anthocyanins (via UDP-flavonoid glucosyl transferase, UFGT) [35]. Previous studies on *P. persica* identified some key structural genes from the flavonoid pathways regulating coloration. The gene CHS (chalcone synthase, *ppa006888m*) was pinpointed as an important modulator of the anthocyanin content in *P. persica* [32, 36]. Here, we found that its ortholog *gene26* may also be involved in the modulation of the flavonoid/anthocyanin contents in *P. mira*. In addition to *gene26*, another CHS gene (*gene14431*) was also detected but its expression level was lower as compared to *gene26*. Zhang et al. [37], by investigating the differentially expressed genes controlling the anthocyanin levels in various pigmented nectarines, observed that *ppa025745m* which is the ortholog of *gene14431* was the main candidate gene. We further identified the gene CHI (*gene7875*) which is highly expressed in the blood-flesh fruit type with 2.6 and 3-fold increase in expression level compared to the milk-white and yellow flesh fruit types, respectively. In agreement with our findings, the gene *ppa011276m* from *P. persica* which is the ortholog of *gene7875*, was proposed as an important structural gene regulating the red pigmentation in peach flower [38]. Moreover, the gene UFGT (*gene8838*) which is the ortholog of *ppa005162m* in *P. persica* and previously demonstrated to differentially regulate the anthocyanin accumulation in deeply colored and light-pigmented peach cultivars was also revealed in this study. Importantly, *ppa005162m* was more expressed in the deeply colored cultivar than in the light pigmented one [32, 39], similarly as *gene8838* which displayed an increase in its expression level by 6752- and 35- fold in PMFR and PMHY, respectively, compared to PMHF. Our result perfectly matches the conclusions of Wu et al. [40] who proposed UFGT as the key gene for the red color in *Prumus mume* flower. Other important flavonoid biosynthetic structural DEGs detected in this study and which may affect the anthocyanin levels in *P. mira* are PAL (*gene19650*), F3H (*gene4823* and *gene22560*), C4H (*gene17724*) and 4CL (*gene788* and *gene22193*). Their orthologs were also found to regulate pigmentation in *P. persica* [20, 32, 38]. Overall, most of these identified structural genes involved in the regulation of the flesh coloration in *P. mira* are mapped to early committed steps on the flavonoid biosynthetic pathway and interestingly, they were all highly expressed in the blood flesh fruit, moderately in the yellow flesh fruit and very low in the milk-white flesh fruit, implying that the high activity of these genes lead to a higher anthocyanin content, thus, to a darker pigmentation. Our results corroborate well the conclusions of Chen et al. [38] who also reported that low expression levels of C4H, CHS and F3H in white petals, contrarily to the red petals of peach, reduce the formation of dihydro-kaempferol (DHK), and thereby inhibit the anthocyanin accumulation. In the same line, Jiao et al. [20] also showed that the *PAL* gene was weakly expressed in the white-flesh peach and suggested that the *PAL* gene may be limiting in anthocyanin production, resulting in the white-flesh coloration.

The activity of the structural genes in the flavonoid biosynthetic pathway is regulated by other genes such as transcription factors from the families of MYB, bHLH and WD40 [41]. Here, we identified 5 MYB and 3 bHLH genes that may be the central regulators of the expression of structural genes in the flavonoid biosynthetic pathway, leading to differential accumulation of anthocyanins. Some orthologs (*ppa010277m*, *ppa017136m* and *ppa006295m*) of some of these genes in *P. persica* were previously confirmed to play similar function [38, 39]. Another important finding from this study is the gene *gene23924*, the ortholog of *ppa007748m* in *P. persica*, which encodes a peroxidase (POD) enzyme. The gene *gene23924* is the only differentially expressed gene between the 3 fruit types from the phenylpropanoid pathway which was up-regulated in PMHF (~ 10-fold increase) compared to PMHR and PMHY. Several studies have demonstrated that POD is involved in the anthocyanin degradation in various plant species such as litchi, grape, plum, *Brunfelsia calycina* [42–45]. Hence, our result implies that besides the down-regulation of many structural genes in the phenylpropanoid-flavonoid pathways caused by some MYB and bHLH transcription factors, the degradation of anthocyanins through the high activity of *gene23924* could also be another important mechanism for the milk-white coloration obtained in PMHF.

Our metabolic profiling highlighted five key anthocyanins involved in the differential flesh coloration in *P. mira* fruit. The combinations and levels of accumulation of these compounds may confer the visible colorations. In particular, we observed that PMHR strongly accumulated various anthocyanins, PMHY had moderate contents of anthocyanins while PMHF contained low concentration of anthocyanins, which correlated well with the expression levels of the key structural genes from the flavonoid pathways in these three fruit types. We also delineated in this study the regulatory network underlying the accumulation of the 5 major anthocyanins involved in fruit flesh coloration in *P. mira*. These results could be harnessed by researchers by utilizing genetic approaches to clarify the mechanism of anthocyanin regulation [46]. Intriguingly, some transcripts involved in the carotenoid biosynthetic pathway were found highly correlated with the anthocynanin compounds. It is probable that the accumulation of some carotenoid compounds together with the anthocyanins provides the final visible flesh coloration in *P. mira* fruit. For example, PMHY mainly accumulated anthocyanins which were reported to give a red coloration [39, 47], then, how exactly its yellow pigment is formed is unclear. Previously, Brandi et al. [18] and Falchi et al. [19] demonstrated that a high carotenoid level was determinant for the yellow-flesh coloration in peach. Unfortunately, the widely-targeted metabolomics approach adopted in this study did not allow us to detect carotenoid compounds. Therefore, we propose that future study should investigate the carotenoids content in *P. mira* fruit and correlate with our transcriptome data to illuminate the part played by carotenoids in fruit flesh coloration.

## Conclusions

In the present investigation, we integrated transcriptome and metabolome analysis to clarify the regulatory network underlying fruit flesh coloration in *Prunus mira*. According to the transcriptome changes in the studied 3 fruit types during the various developmental stages, the determination of fruit flesh pigmentation mainly occurred during the fruit ripening period. We further identified a set of 563 differentially expressed genes associated with fruit flesh pigmentation and other major differentiations between the 3 fruit types. Metabolic profiling during the fruit ripening stage revealed 40 differential metabolites between the 3 fruit types, including 5 anthocyanins, which are suggested to be the key molecules associated with flesh coloration in *P. mira*. The regulatory network constructed based on the detected anthocyanin compounds and their correlated genes not only illuminated the regulatory mechanism underlying their biosynthesis but also provided a framework for understanding the interplay between transcriptional expression and metabolite levels according to the pigmentation in *P. mira* fruit flesh. The findings from this study will benefit molecular breeders in their effort to improve fruit appeal and quality in peach.

## Methods

### Fruit materials

Fruit of wild and semi-wild *Prunus mira* trees displaying different flesh colors: PMHR (blood-colored flesh; wild), PMHY (yellow-colored flesh; wild) and PMHF (milk-white colored flesh; semi-wild) were harvested at the pit-hardening (60 days after bloom) (A), cell enlargement (85 days after bloom) (B) and fruit ripening (95 days after bloom) (C) stages (Fig. 1). All fruits were directly collected from the plants at the above mentioned developmental stages. PMHF is considered a good quality fruit by local farmers leading to a gradual increase of its cultivation. Fruits were randomly collected from 50 years-old individual wild trees and 10 years-old semi-wild tree in the city of Linzhi (26°52′-30°40′ latitude, 92°09′-98°47′, longitude), Nidi village in the southeast of the Tibet autonomous region (China). The collection of *P. mira* samples in the wild was authorized by the Tibet Academy of Agricultural Sciences through the permit number: 20171801016 pt-1. The formal identification of the species was conducted by the corresponding author of this article. Voucher specimens of the fruits are deposited to the germplasm collection bureau of the Institute of Vegetables, Tibet Academy of Agricultural and Animal Husbandry Sciences, under the codes: 2X51PM201729, 2X51PM201730 and 2X51PM201731. Then, the fruits were mixed and three biological replicates were sampled, representing in total 27 samples. Flesh samples of the fresh fruits were dissected using a blade, frozen immediately in liquid nitrogen in the field, transported to the laboratory and then stored at − 80 °C until further use.

### Transcriptome sequencing

Nine libraries based on samples representing the three fruit flesh colors and the three developmental stages were constructed for transcriptome sequencing. Total RNAs were extracted using TRIzol reagent (TaKaRa, Dalian, China) according to the manufacturer’s instructions, and the mRNAs were enriched using magnetic oligo (dT) beads. The quantity and quality of mRNAs were assessed by ND-1000 Nanodrop spectrometer (Nanodrop Technologies, USA) and the Agilent 2100 Bioanalyzer (Agilent Technologies, USA). The mRNAs were added to a fragmentation buffer to break into short fragments. The cDNAs were synthesized with a six base random primer and then, AMPure XP beads were use to purify the double-stranded cDNA. The purified double-stranded cDNAs were first repaired at the end, a tail was added, the sequencing connector connected, and the fragment size was selected using the AMPure XP beads. Finally, PCR amplification was performed and a chain-specific cDNA library was obtained by purifying PCR products with the AMPure XP beads. Sequencing was performed following standard Illumina methods and protocols. The cDNA libraries were sequenced on an Illumina Hiseq platform and 150 bp single-end reads were generated at the Mega Genomics Co., Ltd. (www.megagenomics.cn).

### Transcriptome data analysis

The sequencing reads containing low-quality (containing > 50% bases with a Phred quality score < 15), more than 1% ambiguous residues (N) and adaptor sequences were removed before downstream analyses using the FastQC tool (http://www.bioinformatics.babraham.ac.uk/projects/fastqc/). After filtering, the remaining high-quality reads “clean reads” stored in FASTQ were used for statistical analyses. The clean reads were then mapped to the *P. persica* reference genome (ftp://ftp.ncbi.nlm.nih.gov/genomes/all/GCF/000/346/465/GCF_000346465.2_Prunus_persica_NCBIv2/GCF_000346465.2_Prunus_persica_NCBIv2_genomic.fna.gz) [27] using the TopHat2 tool [48]. The genomic localization results of all sequencing reads data were assembled with the cufflinks package [49], and then were compared to the known genetic models in order to identify new genes and alternative splicing events using the cuffcompare package [49]. We used the blast software [50] and the nr [51], swiss-prot [52], GO [53], KEGG [54] databases to obtain annotated information about the new genes. The gene expression level was determined according to the number of fragments per kilobase of exon per million fragments mapped (FPKM). The EBSeq program [55] was used to perform the differential expression analysis with fold change (FC) ≥ 2 or FC ≤ 1/2 and false discovery rate (FDR) < 0.01 set as screening criteria. Gene ontology (GO) annotation and kyoto encyclopedia of genes and genomes (KEGG) pathway analyses were conducted using the Blast2GO software [56].

### Metabolic profiling

The sample preparation, extract analysis, metabolite identification and quantification were performed at Wuhan MetWare Biotechnology Co., Ltd. (www.metware.cn) following their standard procedures and previously described by Wang et al. [57] and Cao et al. [58].

### Metabolite data analysis

First, a quality control (QC) analysis was performed to verify the reliability of the data. Sample extracts were mixed and inserted into every 10 samples to monitor the changes in repeated analyses. Metabolomics data have the characteristics of high dimension dataset, so it is necessary to combine univariate and multivariate statistical analyses to accurately excavate the differential metabolites. Statistical analyses were performed using the Analyst 1.6.1 software (AB SCIEX, Ontario, Canada). Statistical significance and fold change of the metabolites between the samples were tested with two-paired *t* test. The supervised multivariate method, partial least squares-discriminant analysis (PLS-DA), was used to maximize the metabolome difference between the developmental stages, as well as the difference between the three flesh colored fruits. The relative importance of each metabolite to the PLS-DA model was evaluated using the variable importance in projection (VIP). Metabolites with VIP ≥ 1 and fold change ≥2 or fold change ≤0.5 were defined as differentially accumulated metabolites between compared samples [57, 58].

### Integrative analysis of metabolome and transcriptome datasets

For the joint analysis between the metabolome and transcriptome datasets, the mean of all biological replicates of differential metabolites in the metabolome data and the mean value of expression of differential transcripts in the transcriptome data were calculated. Next, the log_2_ transformed datasets were loaded in the ‘*cor*’ package from the R software (www.r-project.org/). The Pearson correlation (*r*) between metabolites and transcripts was represented by network diagrams, and the differential genes and metabolites between PMHF, PMHR and PMHY were selected when *R*^2^ > 0.9 [46]. Metabolome and transcriptome relationships were visualized using the Cytoscape software version 3.6.1 [59].

### qRT-PCR expression profiling of selected genes

RNAs from flesh samples were extracted using the EASYspin Plus kit (Aidlab Biotechnologies, China) according to the manufacturer’s instructions. The RNA was treated with DNaseI and 1 μg RNA was reverse transcribed with oligo (dT23) primer using the FastQuant RT kit (Tiangen Biotech, China) in a final volume of 25 μL. The specific primer pairs of the ten selected genes were designed using the Primer5.0 software [60] and presented in Additional file 18: Table S13. The qRT-PCR analysis was performed as described by Dossa et al. [61] using the ChamQ SYBR qPCR Master Mix (Vazyme Biotec, China) on a Light Cycler 480 II (Roche, Switzerland). Each reaction was performed using a 20 μL mixture containing 10 μL of 2× ChamQ SYBR qPCR Master Mix, 6 μL of nuclease-free water, 1 μL of each primer (10 mM), and 2 μL of 4-fold diluted cDNA. All of the reactions were run in 96-well plates and each cDNA was analyzed in triplicate. The following cycling profile used: 95 °C for 30 s, followed by 40 cycles of 95 °C/10 s, 60 °C/30 s. The relative expression levels of the selected genes were normalized to the expression level of the ACTIN gene [62]. This analysis was carried out in three independent biological replicates and three technical replicates of each biological replicate.

## Supplementary information

**Additional file 1: Table S1.** Overview of the transcriptome sequencing dataset and quality check in 3 *Prunus mira* fruit types (PMHF, PMHR and PMHY) during 3 developmental stages (pit-hardening (A), cell enlargement (B) and fruit ripening (C)). (XLSX 10 kb)

**Additional file 2: Table S2.** Full list of the unique genes detected in *Prunus mira* fruit transcriptome and their functional annotation. (XLSX 2092 kb)

**Additional file 3: Table S3.** List of the novel genes specific to *Prunus mira* detected from fruit transcriptome and their functional annotation. (XLSX 109 kb)

**Additional file 4: Table S4.** List of the optimized genes based on gene structure optimization analysis in *P. persica. (XLSX 301 kb)*

**Additional file 5: Table S5.** Intron-exon structure of the optimized genes in *P. persica*. (XLSX 3036 kb)

**Additional file 6: Table S6.** List of all the differentially expressed genes identified in this study along with their FPKM data. (XLSX 4901 kb)

**Additional file 7: Figure S1.** KEGG enrichment analysis of the 343 genes constantly and differentially expressed during the fruit development and ripening in *Prunus mira*. (TIF 522 kb)

**Additional file 8: Figure S2.** KEGG enrichment analysis of the 3736 genes constantly and differentially expressed during the transition from the pit-hardening to the cell enlargement stages in *Prunus mira*. (TIF 565 kb)

**Additional file 9: Figure S3.** KEGG enrichment analysis of the 607 genes constantly and differentially expressed during the transition from the cell enlargement to the fruit ripening stages in *Prunus mira*. (TIF 556 kb)

**Additional file 10: Figure S4.** qRT-PCR (2^-ΔΔct^) analysis of 10 selected genes within the differentially expressed genes detected in this study. Correlation analysis between qRT-PCR and RNA-seq (log2fold change). (TIF 886 kb)

**Additional file 11: Table S7.** List of the differentially expressed genes between the 3 *Prunus mira* fruit types (PMHF, PMHR and PMHY) representing the ‘*core transcriptome*’ and their functional annotation. (XLSX 26 kb)

**Additional file 12: Figure S5.** KEGG enrichment analysis of the 563 genes differentially expressed in PMHF, PMHR and PMHY during the fruit ripening stage, representing the ‘*core transcriptome*’ in *Prunus mira*.s (TIF 522 kb)

**Additional file 13: Table S8.** List of the differentially expressed genes between the 3 *Prunus mira* fruit types (PMHF, PMHR and PMHY) mapped to the terpernoid-carotenoid pathways and phenylpropanoid-flavonoid pathways and their expression level during the fruit ripening stage (C). (XLSX 11 kb)

**Additional file 14: Table S9.** List of the bHLH and MYB transcription factors detected in the ‘*core transcriptome*’ and their expression fold change in group comparisons between the 3 *Prunus mira* fruit types (PMHF, PMHR and PMHY) during the fruit ripening stage. (XLSX 10 kb)

**Additional file 15: Table S10.** Overview of the metabolites detected and quantified in 3 fruit types of *Prunus mira* (PMHF, PMHR and PMHY) during 3 developmental stages (pit-hardening (A), cell enlargement (B) and fruit ripening (C)). Data are from 3 biological replicates and the mix01 to mix05 represent the mixture of sample extracts. (XLSX 138 kb)

**Additional file 16: Table S11.** List of the differential accumulated metabolites between the 3 *Prunus mira* fruit types (PMHF, PMHR and PMHY) representing the ‘*core metabolome*’ and their expression fold change between group comparisons during the fruit ripening stage. (XLSX 15 kb)

**Additional file 17: Table S12.** List of genes showing a high correlation (R^2^ > 0.9) with the 5 anthocyanins detected as important molecules involved in the fruit flesh coloration in Prunus mira*. (XLSX 68 kb)*

**Additional file 18: Table S13.** The primer sequences for real time PCR. (XLSX 9 kb)

## Data Availability

All data generated or analysed during this study are included in this published article and its supplementary information files. The raw RNA-seq data are freely available at: www.ncbi.nlm.nih.gov/bioproject/PRJNA510449. The annotated gene file and files containing the gene sequences in fasta are available at Figshare: https://figshare.com/articles/Prunus_mira_gene_annotation_file/9598799 and https://figshare.com/articles/Prunus_mira_genes_in_fasta_format/9598808. File containing the gene sequences in fasta has also been deposited at DDBJ/EMBL/GenBank under the accession GHVK00000000. The version described in this paper is the first version, GHVK01000000.

## Abbreviations

4CL: 4 coumarate CoA ligase
ANS: Anthocyanidin synthase
bHLH: basic helixloop-helix
C4H: Cinnamic acid 4-hydroxylase
CHI: Chalcone isomerase
CHS: Chalcone synthase
DAM: Differentially accumulated metabolites
DEG: Differentially expressed genes
DFR: Dihydroflavonol 4-reductase
DHK: Dihydro-kaempferol
F3H: Flavonone 3-hydroxylase
FC: Fold change
FPKM: Fragments per kilobase of exon per million fragments mapped
GO: Gene ontology
HCA: Hierarchical cluster analysis
KEGG: Kyoto encyclopedia of genes and genome
LDOX: Leucoanthocyanidin dioxygenase
PAL: Phenylalanine ammonia-lyase
PCA: Principal component analysis
PLS-DA: Partial least squares-discriminant analysis
POD: Peroxidase
QC: Quality control
UFGT: UDP-glucose-flavonoid 3-*O*-glucosyltransferase
VIP: Variable importance in projection
