# Supplementary figures and images for: Transcriptomic and metabolomic profiling provide novel insights into fruit development and flesh coloration in Prunus mira Koehne, a special wild peach species
Source: BMC Plant Biol. 2019 Nov 1;19:463. doi: 10.1186/s12870-019-2074-6 (PMC6825364; doi:10.1186/s12870-019-2074-6)

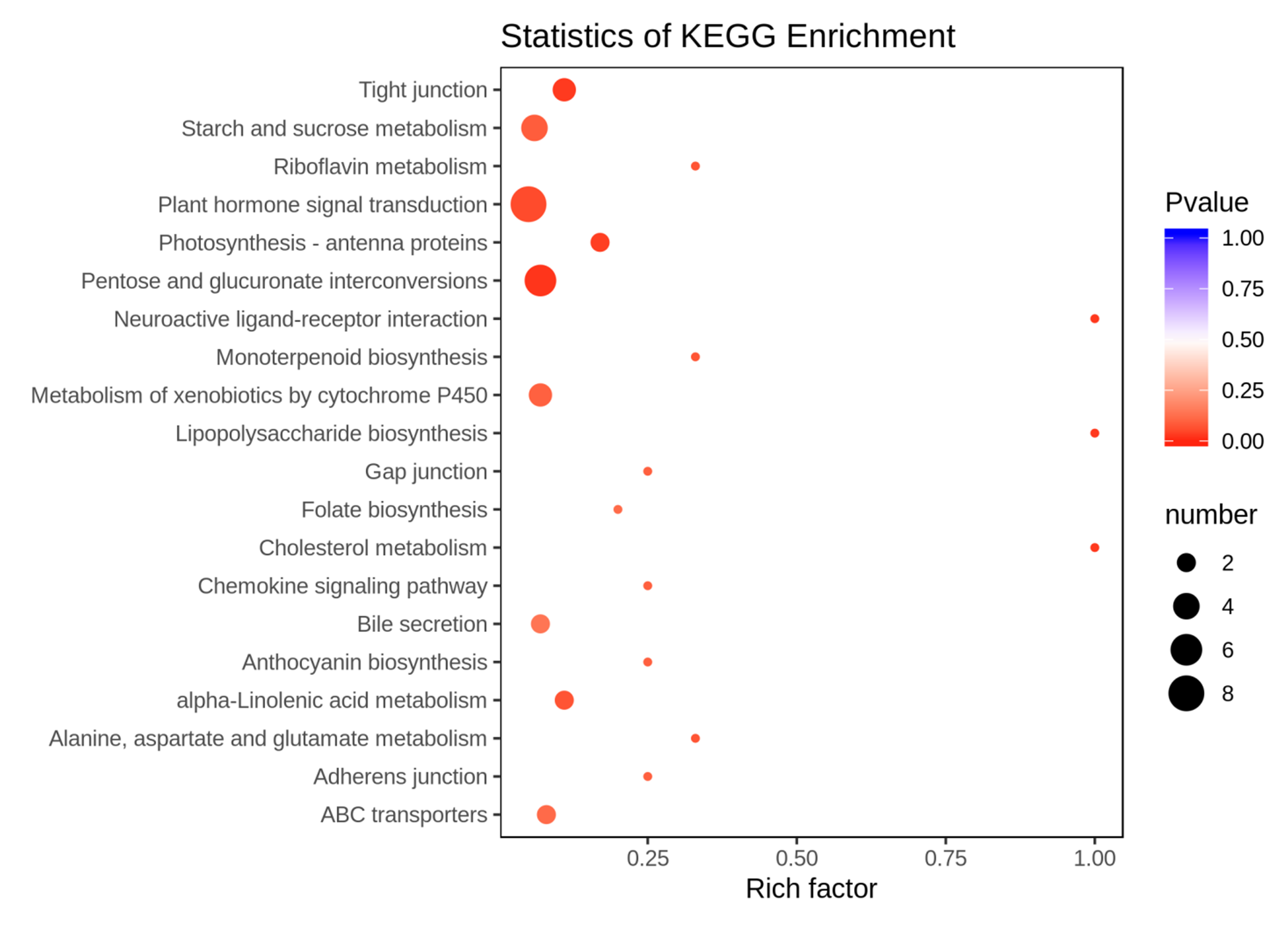

Supplement: Supplementary file 7 — Additional file 7: Figure S1. KEGG enrichment analysis of the 343 genes constantly and differentially expressed during the fruit development and ripening in Prunus mira. (TIF 522 kb) [file 12870_2019_2074_MOESM7_ESM.tif]

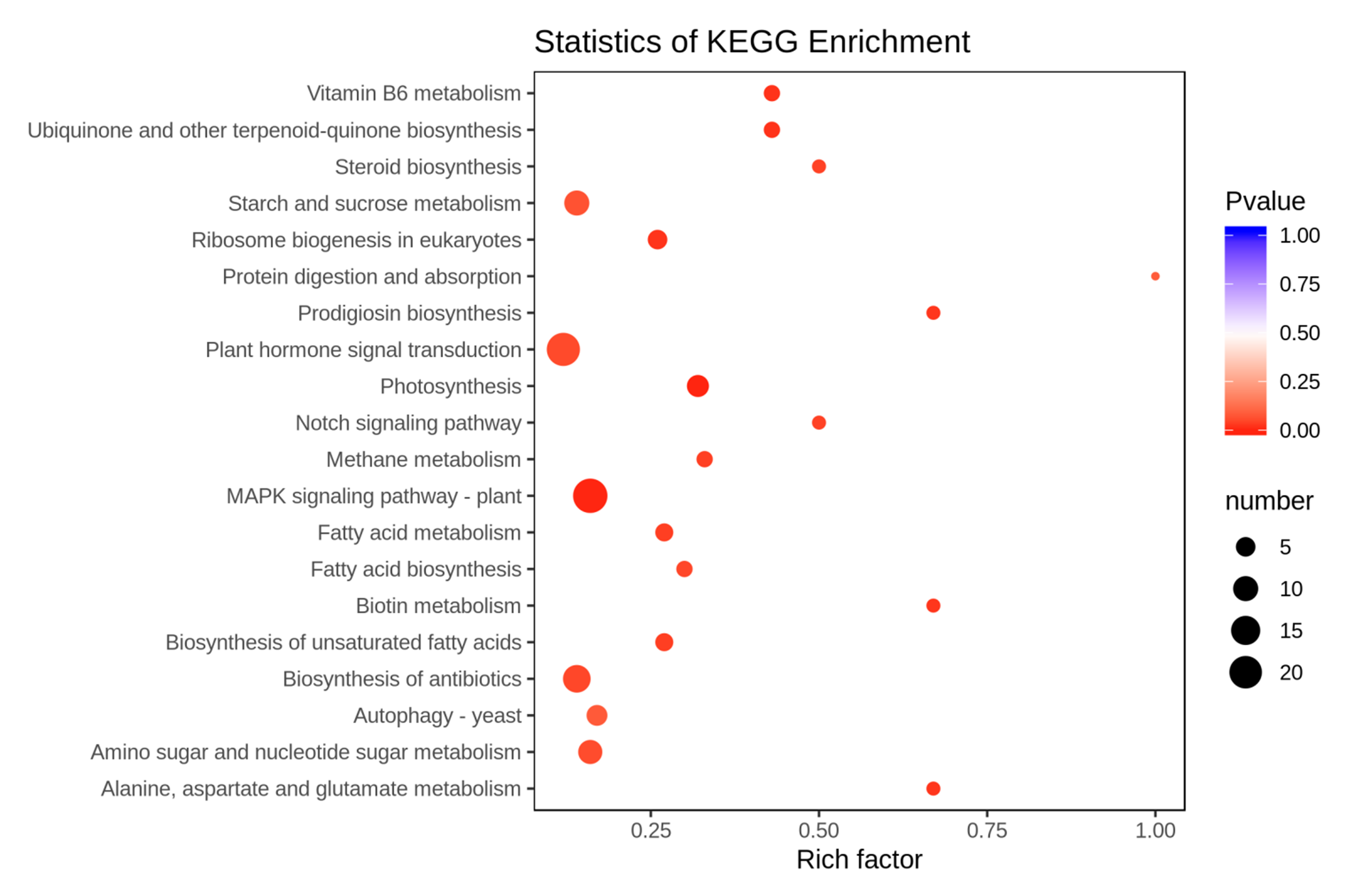

Supplement: Supplementary file 8 — Additional file 8: Figure S2. KEGG enrichment analysis of the 3736 genes constantly and differentially expressed during the transition from the pit-hardening to the cell enlargement stages in Prunus mira. (TIF 565 kb) [file 12870_2019_2074_MOESM8_ESM.tif]

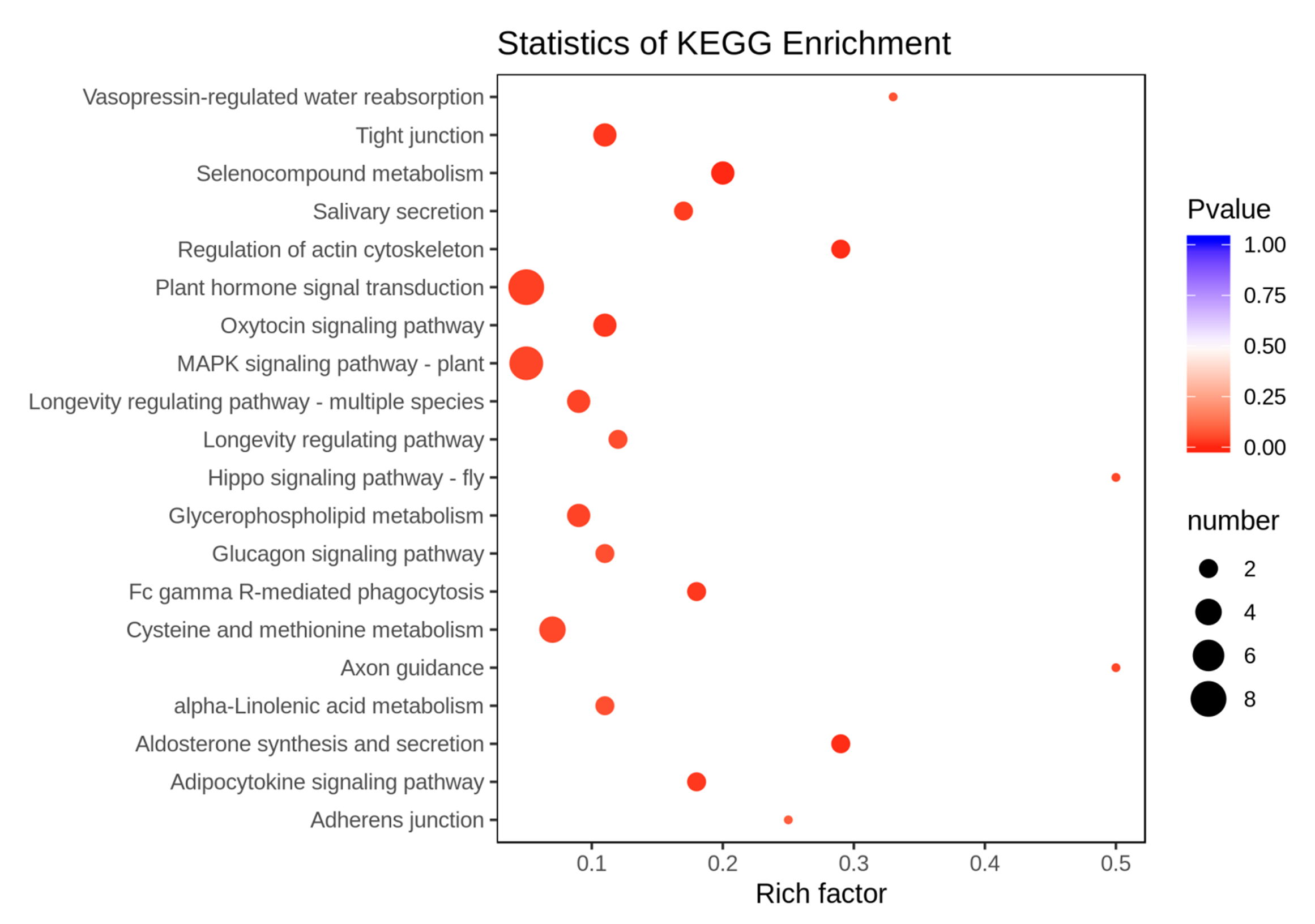

Supplement: Supplementary file 9 — Additional file 9: Figure S3. KEGG enrichment analysis of the 607 genes constantly and differentially expressed during the transition from the cell enlargement to the fruit ripening stages in Prunus mira. (TIF 556 kb) [file 12870_2019_2074_MOESM9_ESM.tif]

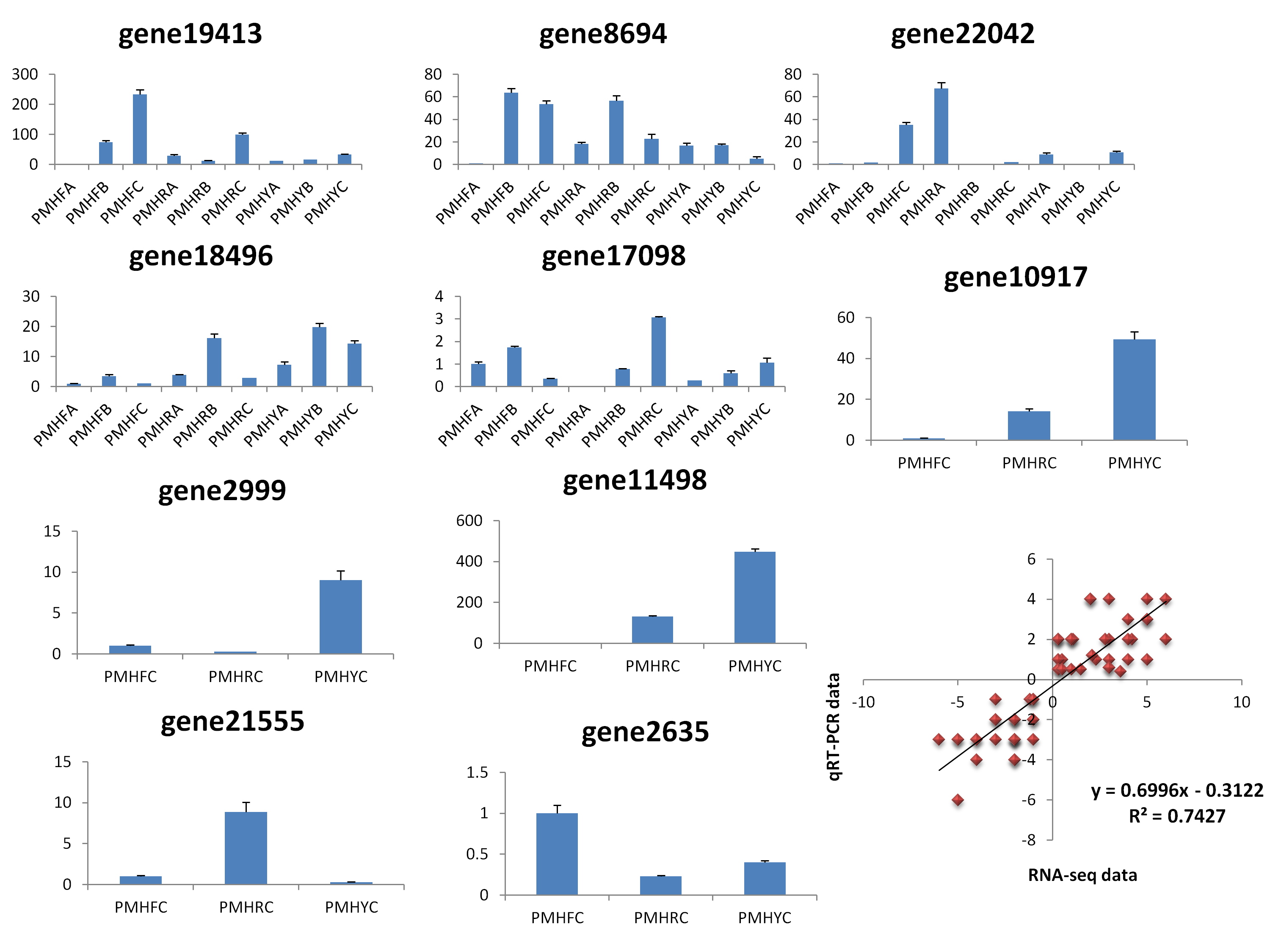

Supplement: Supplementary file 10 — Additional file 10: Figure S4. qRT-PCR (2-ΔΔct) analysis of 10 selected genes within the differentially expressed genes detected in this study. Correlation analysis between qRT-PCR and RNA-seq (log2fold change). (TIF 886 kb) [file 12870_2019_2074_MOESM10_ESM.tif]

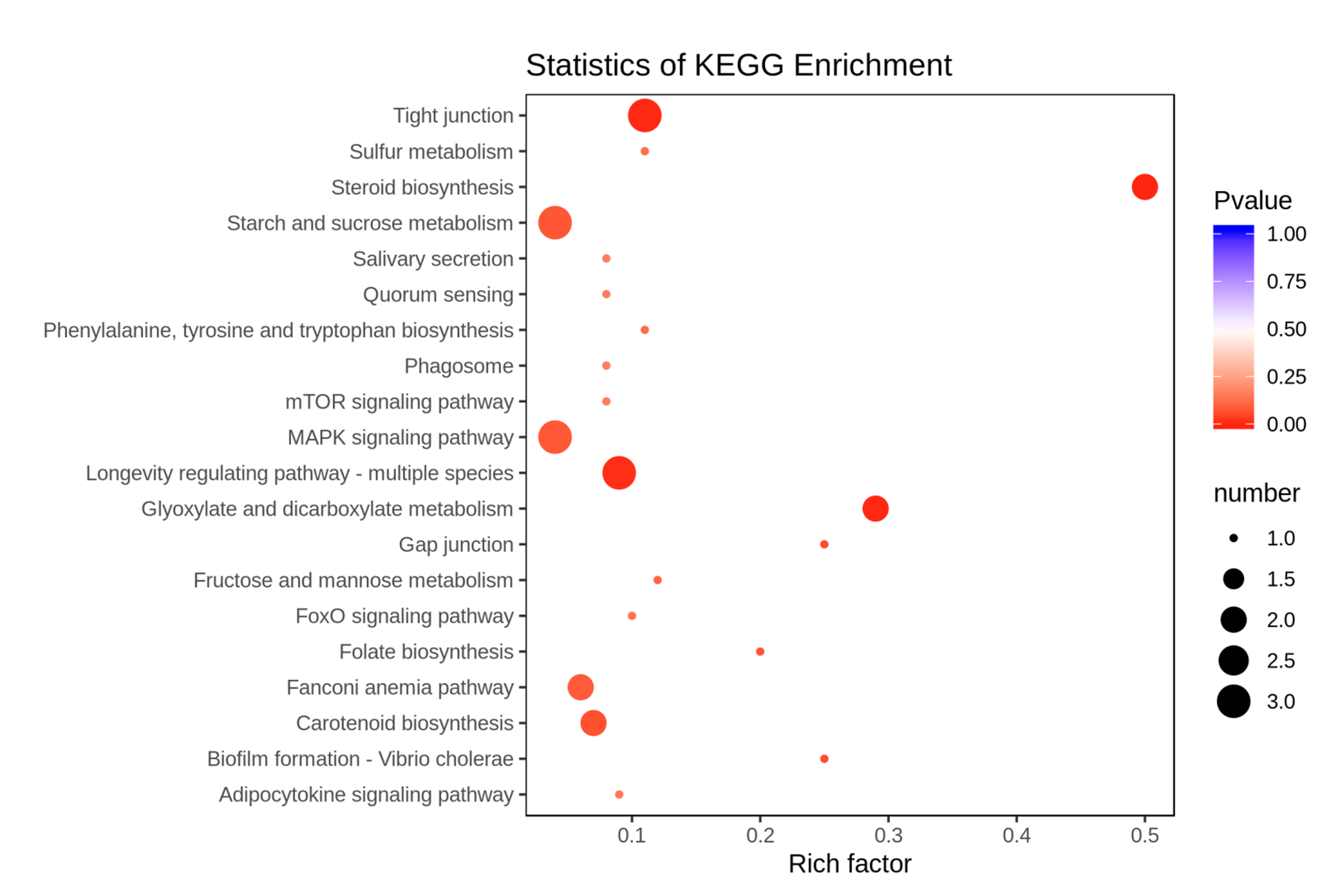

Supplement: Supplementary file 12 — Additional file 12: Figure S5. KEGG enrichment analysis of the 563 genes differentially expressed in PMHF, PMHR and PMHY during the fruit ripening stage, representing the ‘core transcriptome’ in Prunus mira.s (TIF 522 kb) [file 12870_2019_2074_MOESM12_ESM.tif]
